# Alkyl-carbon chain length of two distinct compounds and derivatives are key determinants of their anti-*Acanthamoeba* activities

**DOI:** 10.1038/s41598-020-62934-8

**Published:** 2020-04-14

**Authors:** Ronnie Mooney, Mariana Masala, Theo Martial, Charles McGinness, Fiona Luisa Henriquez, Roderick Adeyinka Malcolm Williams

**Affiliations:** Institute of Biomedical and Environmental Health Research, University of the West of Scotland, School of Health and Life Sciences, High Street, Paisley, PA1 2BE Scotland UK

**Keywords:** Phenotypic screening, Parasitic infection

## Abstract

The opportunistic pathogen, *Acanthamoeba castellanii* is the causative agent for the sight threatening infection *Acanthamoeba* keratitis (AK). It is commonly associated with contact lens wearers, and prevalence is increasing at an alarming rate due to an inadequate preventive strategy to protect the lens from this protist. This problem is compounded by the lack of an effective acanthamoebocide, particularly with cysticidal activity in the contact lens solutions. We have used cytotoxicity assays and a variety of biophysical approaches to show that two molecules with tails made of alkyl carbon, alkylphosphocholines (APCs) and quaternary ammonium compounds (QACs) had significant chain-length dependent efficacy against *A. castellanii* trophozoites, the latter producing death via permeabilization, and DNA complexing. QACs were more effective than APCs and had activity against cysts. Conversely, the QAC with 12 alkyl carbon chain, was non toxic, its presence increased *A. castellanii* trophozoites biomass and delayed encystation by 96 h. Interestingly, it was unable to induce excystation and increased trophozoite sensitivity to APC16. These results present a mono- and multi-inhibitor management strategy effective against trophozoites and cysts that may be useful for formulating into contact lense cleaning solutions and reducing AK incidence.

## Introduction

*Acanthamoeba* are free-living protists, existing as active trophozoites and non-replicative cysts characterised by double cellulose cell walls^1^. They have opportunistic tendencies and can cause the diseases, *Acanthamoeba* keratitis (AK), cutaneous acanthamoebiasis (CA) and granulomatous amoebic encephalitis (GAE)^2,3^. Whilst CA and GAE are lethal but rare and affect the skin and CNS, respectively, the non-lethal AK causes severe infection in the cornea, threatening sight loss and causing emotional and psychological trauma, described as life-changing in patients^4^. Trophozoites to cysts interconversion and vice versa (encystation and excystation) is induced by external environmental cues^5,6^ and poses significant challenges for curative treatment particularly causing AK resurgences and relapses^7^.

AK cases are increasing and prevalence is high amongst contact lens users^8,9^. Cases of AK within the UK have risen drastically within the last 20 years and continue to do so^10^. The reason for this upsurge in incidence rates does not appear to be as a result of increased lens wearers but is as of yet unknown, likely the result of multiple factors^10^. Spatial geographic differences are common with 15 times more cases reported in the UK than the USA^11,12^, and 8 times more in Scotland than England^13^. These differences are strongly linked with water supply, quality and usage^12^. Indeed, the incidence of AK in contact lens users is believed to be due to rinsing of contact lenses with domestic tap water^14^. Further, the lack of anti-acanthamoebocides in cleaning solutions is another issue. Despite the presence of preservative compounds such as polyhexamethylene biguanide used in existing solutions, these are ineffective at killing cysts and induce encystation^15,16^. AK prevention thus requires an improved contact lens cleansing formulation for an effective preventive strategy.

The identification of compounds with anti-acanthamoebic properties particularly against cysts is challenging as individual drugs are mainly ineffective^15^. Established or experimental drug combinations have multiple molecular targets. For example, in the clinic combinations of polyhexamethylene biguanide (PHMB), chlorhexidine, propamidine isethionate, dibromopropamidine and hexamidine are used^17^. Experimental compounds to prevent or delay encystation during treatment with encystment inhibitors specific to encystation metabolic pathways e.g. autophagy^18,19^, serine and cysteine proteases and cellulose biosynthesis^20,21^. This causes *Acanthamoeba* to persist long enough as the vulnerable trophozoites^22,23^ and to allow a second cytotoxic agent to exert its effect^24^. The combination of different administration routes has also provided positive outcomes, for example topical and systemic administration using topical chlorhexidine 0.06% and propamidine isetionate 0.1% and the zwitterionic alkylphosphocholine (APC) called miltefosine^25,26^. APCs are also effective against kinetoplastids (*Trypanosoma* and *Leishmania*), anaerobic parasites (*Trichomonas* and *Entamoeba*) and other amoebae (*Naegleria*, as well as *Acanthamoeba*) trophozoites and cysts, but can induce encystation in *Acanthamoeba*^27–32^. Structure-activities studies in *Leishmania*^33^ and *Acanthamoeb*a^29^ have demostrated that changes in the physical strucure of the APC can increase efficacy. Herein, we present a comparative structure-activity study of cationic quaternary ammonium compounds (QACs) and APCs. Our results showed that the alkyl carbon chain lengths (14–18 carbons) and overall change of the molecule were the main determinant for their anti-acanthamoebic activity against *A. castellanii*, producing death by leakage and DNA complexing. Efficacy for APCs was lower than QACs. One QAC with 12 alkyl carbon chain (QAC12), when used alone against tropohzoites was non toxic. In contrast, it was used as an energy substrate, increased biomass and delayed encystation. Conversely, QAC12 in combination with APC16 was synergistic against trophozoites likely due to the delay in encystation. The results present different strategies for an effective preventive treatment strategy for *Acanthamoeba*.

## Results

### Long and short alkyl-carbon chain QACs have contrasting activities against *A. castellanii* trophozoites

**Figure 1:**
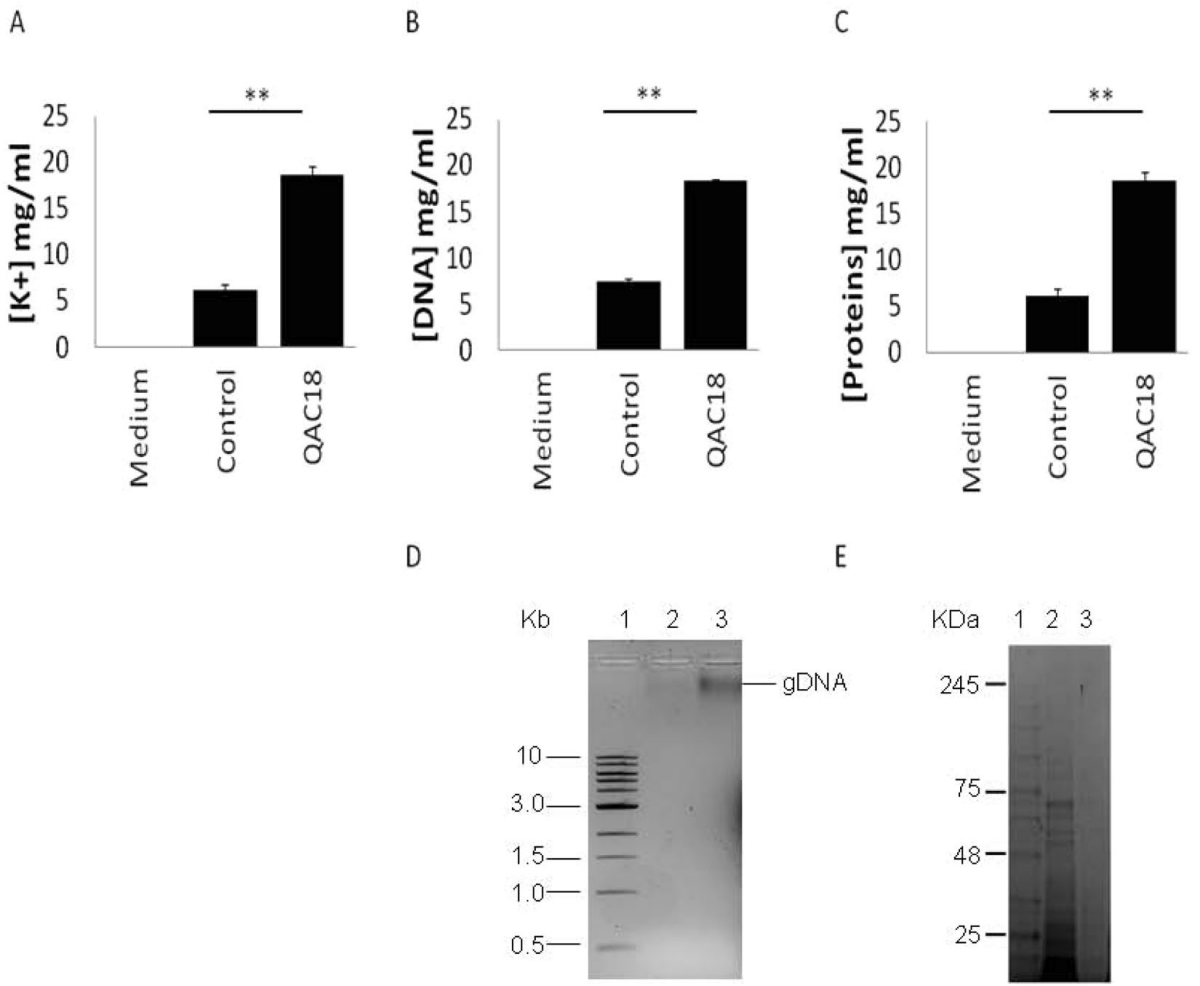
Extrusion of cytoplasmic constituents of *A. castellanii* trophozoites to the external milieu after QAC treatment. [K^+^] (**A**), [DNA] (**B**) and [proteins] (**C**) released from 10^5^ *A. castellanii* trophozoites after treatment without (control) and with QAC18 at 37.5 µg/ml for 24 h into spent medium and measured with the Atomic Absorption Spectroscopy (**A**), the Nanodrop at 260 nm (**B**) by Bradford assay (**C**) respectively were increased 3.6-fold, 4.1-fold and 15.1-fold respectively. Data is mean ± SD; n = 4 independent experiments performed in triplicates. **Student’s t-test showed significant difference of [DNA], [proteins] and [K+] in the spent media of control and QAC18-treated cells at p < 0.01. Extracellular DNA was visualised on 0.75% agarose gel (**D**), Key: Lane 1; DNA ladder, Lane 2; spent media on untreated trophozoites, Lane 3; spent media of QAC18-treated trophozoites and extracellular proteins visualised on SDS-PAGE (**E**). Key; Lane 1; protein ladder, Lane 2; spent media of QAC18-treated trophozoites, Lane 3; spent media on untreated trophozoites.

**Figure 2:**
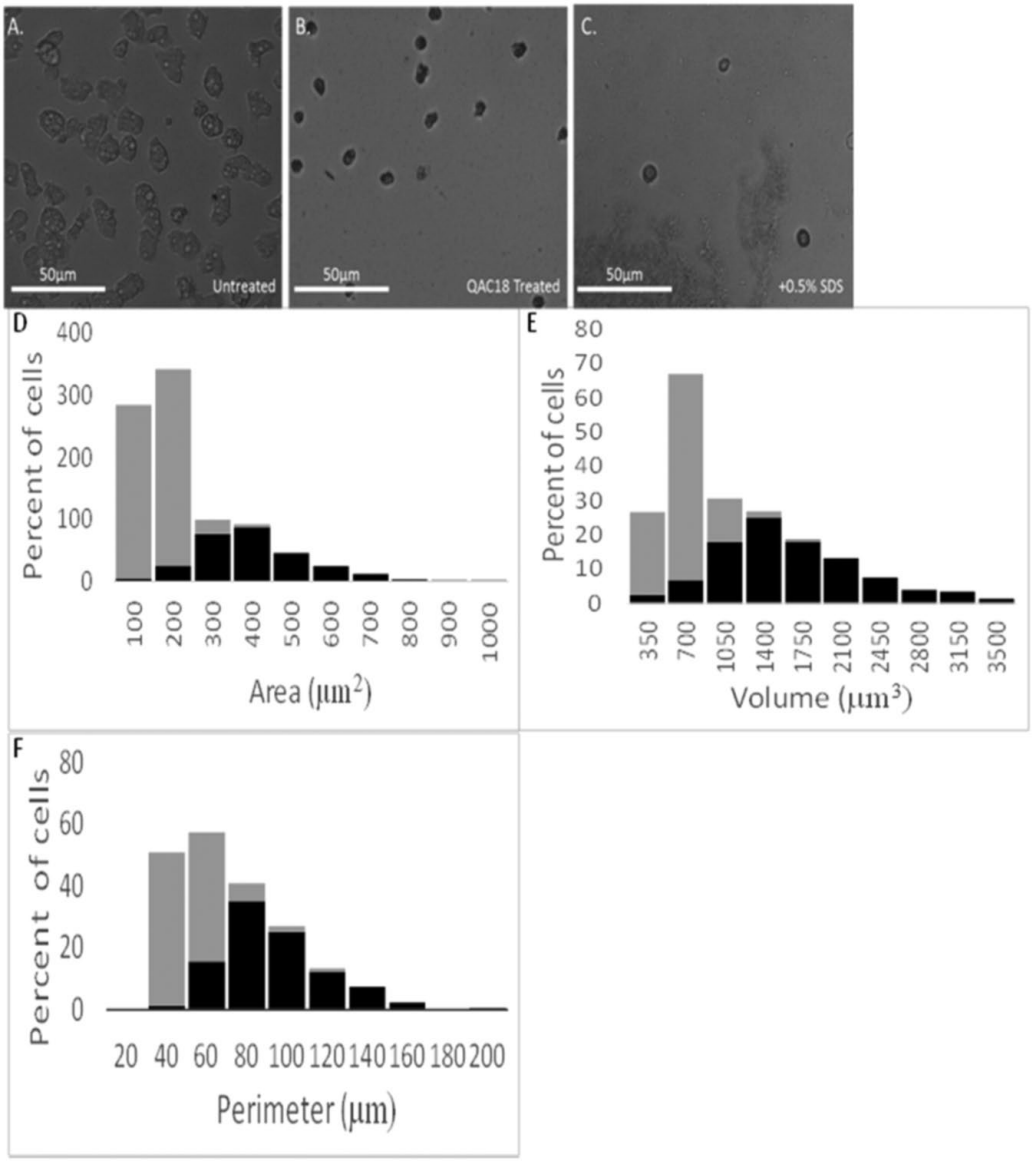
QAC18 induced a cyst-like morphology in *A. castellanii*. *Acanthamoeba* trophozoites (10^5^ cells/ml) incubated without (**A**) with QAC18 at 37.5 µg/ml for 1 h at 25 °C (**B**) and 0.5% SDS for 30 min (**C**). QAC18 reduced trophozoites sizes from 29.02 ± 0.23 (**A**) to 7.87 ± 0.93 (**B**) and disintegrated after 30 min treatment with SDS (**C**). Distribution of the area (µm^2^) (**D**), optical volume (µm^3^) (**E**) and perimeter length (µm) (**F**) of trophozoites treated for 24 h with (grey bar) and without (black bar) QAC18 assessed using the Holomonitor M4.

**Figure 3:**
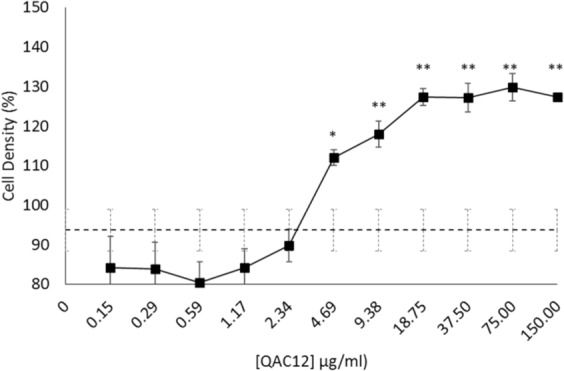
QAC12 increased *A. castellanii* cell density *in vitro*. *Acanthamoeba* trophozoites (10^5^ cells/ml) treated with 150 µg/ml QAC12 doubly serially diluted to 0.015 µg/ml for 96 h at 25 ^o^C. Cell viability estimated by the alamar blue assay and expressed as a percentage showed dose-dependent increase in mean absorbance, a measure for cell density, which was significant at 9.38 µg/ml and above, a 23% increase in cell density relative to control cells without QAC12 was observed at this concentration, with density peaking at 37.5 μg/ml and above (>32% increase). Average cell density of untreated cells is 94% (dashed line). Students t-test p < 0.01 as denoted by “*”, data is mean viability ± SD; n = 6 independent experiments.

*A. castellanii* trophozoites presented contrasting responses to the cationic QACs with alkyl-carbon chain lengths ranging from 12–18 (named QAC12-QAC18; Table 1). IC_50_s from QAC14 to QAC18 increased progressively with decreased alkyl-carbon chain lengths (Table 2) with death occurring below their corresponding critical micelle concentration after 96 h (Table 2). QAC18 was the most effective. Leakage of DNA, proteins and K^+^ from *A. castellanii* trophozoites into the external milieu was noted in QAC18-treated cells (37.5 μg/ml) after 24 h. Concentrations of [K^+^], [DNA] and [proteins] in spent medium were elevated 3.6-fold (Figs. 1A), 4.1-fold (Fig. 1B) and 15.1-fold (Fig. 1C) respectively and the extruded DNA and proteins are shown in Fig. 1D,E respectively. Concurrently, the QAC18-treated trophozoites became smaller than their untreated counterpart in width (4-fold; 7.87 ± 0.93 µm and 29.35 ± 0.23 µm; Fig. 2A,B). Morphometric analysis of QAC18 treated and untreated trophozoites of *A. castellanii*, in *in vitro* cultures showed changes in mean area (377.93 ± 151.78 µm^2^ and 112.24 ± 42.66 µm^2^), perimeter (84.66 ± 24.34 µm and 42.81. ± 12.20 µm), and volume (1628.80 ± 734.91 µm^3^ and 508.44 ± 220.05 µm^3^), after 24 h treatment, with smaller trophozoites much more abundant in QAC-treated cells (Fig. 2D,E respectively). The shrunk trophozoites were calcofluor white negative (data not shown) and sensitive to disintegration by 0.5% SDS (92%; Fig. 2C), suggesting that encystation had not occurred. Failure of the shrunk cells to oxidise resazurin after the removal of QAC18 and resuspension in PG medium in a 6 hour incubation assay, suggests that this compounds is toxic; QAC16 was active after 24 h at 18.75 μg/ml while QAC14 was active after 24 h at 37.5 μg/ml (Table 3). QAC12 was inactive against *A. castellanii* trophozoites at up to 150 µg/ml for up to 96 h and surprisingly, produced a dose-dependent increased biomass which suggested that it was an energy substrate (Fig. 3).

**Table 1:** Structures of QACs and APCs.

Alkyl carbon	QACs	APCs
18		
16		
14		
12

**Table 2:** IC_50_s of QACs against *A.castellanii*.

Compound	Abbreviation	No. of carbon	CMC (µM)	MW (g/mol)	IC_50_			
					*A. castellanii* trophozoites		*A. castellanii* cysts	
					µg/ml	µM	µg/ml	µM
Dodecyltrimethyl ammonium bromide	QAC12	12	21300*	308.40	>150	>486	>37.50	>121.60
tetradecyltrimethyl ammonium bromide	QAC14	14	5600*	336.40	52.80 ± 0.14	156.96 ± 0.12	>37.50	>111.47
hexadecyltrimethyl ammonium bromide	QAC16	16	1300*	364.50	10.80 ± 0.34	29.63 ± 0.03	19.17 ± 0.56	52.59 ± 0.64
octadecyltrimethyl ammonium bromide	QAC18	18	200 (800)**	392.50	6.90 ± 0.03	17.58 ± 0.04	15.00 ± 0.34	38.21 ± 0.24

The CMC of QACs collated from *Jalali-Heravi & Konouz, 2003; **Quan *et al*., 2007 (Lukac *et al*., 2013). Data is mean ± SD; n = 4 independent experiments performed in triplicates. Student’s t-test showed significant difference between all treatments for each parasite life cycle stage at p < 0.01.

**Table 3:** Back transformation of QAC-treated cysts to trophozoites after drug wash-out.

Drugs	Drug concentration (µg/ml)	Trophozoites					Cysts		
		Hours							
		1	24	48	72	96	24	48	72
QAC14	37.50	+	+	+	+	+	+	+	+
	18.75	+	+	+	+	+	+	+	+
	9.375	+	+	+	+	+	+	+	+
QAC16	37.50	−	−	−	−	−	+	−	−
	18.75	+	−	−	−	−	+	+	+
	9.375	+	+	+	+	+	+	+	+
QAC18	37.50	−	−	−	−	−	−	−	−
	18.75	+	−	−	−	−	+	−	−
	9.375	+	−	−	−	−	+	+	+
Control	0.0000	+	+	+	+	+	+	+	+

“+”Indicates cells reverting back to trophozoites after washout; and capable of oxidizing reduced alamar blue from blue to pink after 8 days “−” indicates cell that did not revert to trophozoites after wash out and unable to oxidise reduced alamar blue solution

**Figure 4:**
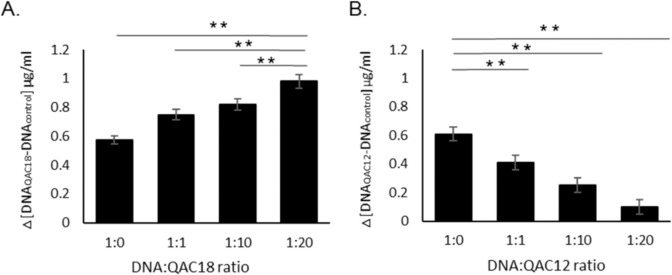
QAC-DNA interaction. The absorbance of genomic DNA (0.6 µg/ml) extracted from *A. castellanii* trophozoites measured at 260 nm with the Nanodrop after incubating for 15 minutes with 0 μg/ml, 0.6 μg/ml, 6 μg/ml or 12 μg/ml of QAC12 (**A**) or QAC16 (**B**) to give ratios of 1:0, 1:1, 1:10 and 1:20. DNA absorbance increased and decreased with increased [QAC18] and [QAC12] respectively. Data is mean absorbance at 260 nm ± SD; n = 4 independent experiments performed in triplicates. Student’s t-test showed significant difference between mean absorbance at 1.1, 1:10 and 1:20 relative to 1:0 at p < 0.01 for QAC18 (**A**) and QAC12 (**B**) respectively.

Examination of the genomic DNA (gDNA) of shrunk QAC18-treated *A. castellanii* trophozoites (18.75 µg/ml) after 96 h, using DNA-agarose gel electrophoresis, the fragmented DNA profile consistent with death via apoptosis was not observed (data not shown). However, gDNA of *A. castellanii* incubated with QAC18 produced a dose-dependent compaction judged by a progressively increased absorbance at 260 nm (Fig. 4A). In contrast, the interaction of QAC12 with *A. castellanii* gDNA produced a dose-dependent decreased absorbance at 260 nm (Fig. 4B). In control experiments, neither QAC12 nor QAC18 had absorption at the concentrations used in the interaction assays.

### Long APCs are toxic to *A. castellanii* trophozoites

The efficacy of the zwitterionic APCs with different alkyl-carbon chain lengths (12–16 carbons) tested against *A. castellanii* was also dependent on tail length, with APC12 and APC16 being the least and most potent with IC_50_s of 32.30 μg/ml and 7.60 μg/ml, respectively (Table 4). The removal of APC16 from treated cells, and incubation in PG medium alone, showed that they were able to oxidise resurzarin after 96 h when treated with up to 37.5 μg/ml, suggesting the compound to be amoebostatic or excystation was occuring (data not shown).

**Table 4:** IC_50_s of APCs against *Acanthamoeba* trophozoites.

Compounds	Abbreviation	No of carbons	MW	IC_50_ (µM)		CMC value (µM)
			g/mol	*Acanthamoeba* trophozoites µg/ml	*Acanthamoeba* trophozoites µM	
Dodecylphosphocholine	APC12	12	351.50	32.3 ± 0.45	91.89 ± 0.34	1000.00*
Tetradecylphosphocholine	APC14	14	379.50	12.7 ± 0.16	33.47 ± 0.24	120.00*
Hexadecylphosphocholine	APC16	16	407.50	7.90 ± 0.26	18.65 ± 0.34	13.00*

Activity of APCs against Acanthamoeba trophozoites incubated with different concentrations of APCs for 72 h and 96 h respectively at 26 °C and toxicity determined using alamar blue^7^. The CMCs for APCs were collated from *Yaseen *et al*. (2005) Biophysical Chemistry 117 263-273 and **Anatrace measurement. N/A- not available Data is mean ± SD; n = 4 independent experiments performed in triplicates. Student’s t-test showed significant difference between all treatments for each parasite at p < 0.01.

### QAC12 delayed encystation

**Figure 5:**
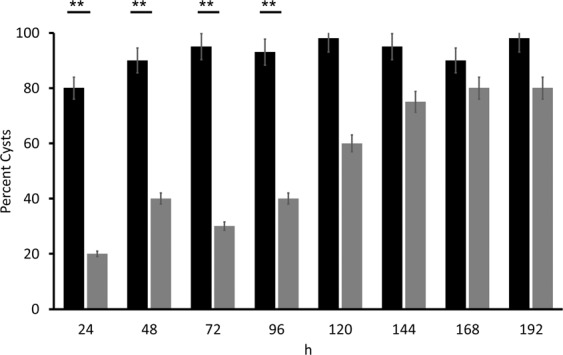
QAC12 delayed *A. castellanii* trophozoite-cyst conversion. *Acanthamoeba* trophozoites (10^6^ cells/ml) incubated for up to 192 h in encystment medium inoculated with (grey) and without (black) QAC12 at 25 ^o^C. The number of cysts produced estimated microscopically and expressed as a percent of total number of cells, trophozoites and cysts. Data are mean ± SD; n = 4 independent experiments performed in triplicates. Student’s t-test showed significant difference between % cysts with (grey bars) and without (black bars) QAC12 added at p < 0.01.

The fact that QAC12 promoted trophozoites growth suggested that it provided a favourable condition for trophozoites and delayed or stop encystation. The incubation of trophozoites in encystment medium, supplemented with 37.5 µg/ml of QAC12 delayed encystation by 96 h (Fig. 5). However, the same concentration of QAC12 was unable to induce excystation of matured cysts (data not shown).

### QAC12 enhanced sensitivity of trophozoites to APC16 but not QAC18

**Figure 6:**
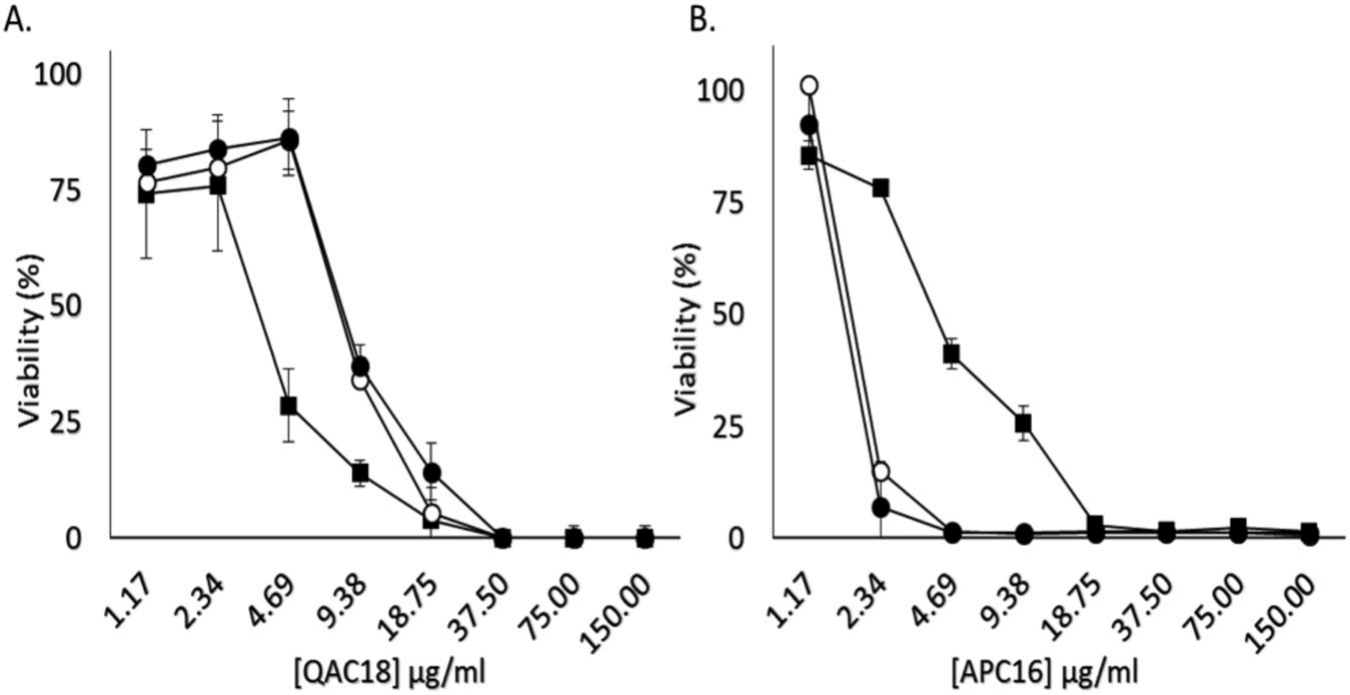
QAC12 increased APC16 efficacy but not QAC18 efficacy *in vitro*. *Acanthamoeba* trophozoites (10^5^ cells/ml. n = 5/treatment) treated with 150 µg/ml doubly diluted to 1.17 µg/ml of QAC18 (**A**) or APC16 (**B**) for 96 h at 25 ^o^C without QAC12 (closed square) and with two concentrations of QAC12 – 18.75 μg/ml (open circle) and 37.5 μg/ml (closed circle). Cell viability estimated using the alamar blue assay showed the addition of QAC12 at both concentrations decreased and increased the potency for QAC18 and APC16 respectively relative to the monotherapies. Data are mean estimated viability ± SD; n = 4 independent experiments performed in triplicates. IC_50_s were statistically significant between both QAC18 and APC monotherapies and the combined treatment using QAC12 at both concentrations (p < 0.01, **A**,**B**).

As QAC12 delayed encystation of *A. castellanii* trophozoites, we postulated that they would be susceptible to the active QACs and APCs. The addition of 37.5 µg/ml or 18.75 µg/ml of QAC12 to QAC18 at concentrations starting from 150 μg/ml doubly diluted 12 times to 1.17  μg/ml produced estimated IC_50_s of 14.6 μg/ml and 11.7 μg/ml respectively, statistically significantly different from QAC18 alone in control experiments (IC_50_ - 6.9 μg/ml; p < 0.01, Fig. 6A).

In contrast, QAC16 combined with APC12 under the same conditions produced shifts from the dose response curves of APC16-treated cells (Fig. 6B). The estimated IC_50_s for APC16 with QAC12 at 37.5 μg/ml and 18.75 μg/ml were 1.74 μg/ml and 2.03 μg/ml, statistically significantly different from the APC16 only in control experiments (IC_50_-7.6 μg/ml; p < 0.01, Fig. 6B). Interestingly, the removal of the inhibition of APC16 at concentrations of 3.12 μg/ml, 4.68 μg/ml, 6.25 μg/ml and 9.36 μg/ml with QAC12 (37.5 μg/ml and 18.75 μg/ml) after 96 h were unable to oxidise resazurin, but their counterpart without QAC12 added were viable, suggesting that the QAC12-APC16 combination increased trophozoites sensitivity to APC16.

### QACs have cysticidal activities against *A. castellanii*. 

**Figure 7:**
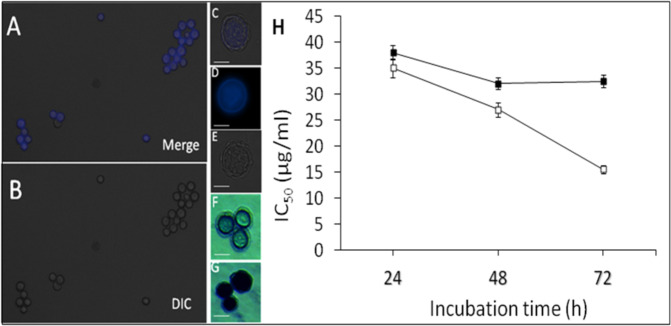
Integrity of *A. castellanii* cyst and cytotoxicity to QACs. *Acanthamoeba* trophozoites (10^5^ cells/ml) incubated in encystment medium for 7 days, stained with calcofluor white for 30 minutes observed with the DAPI **(A**) and DIC (**B**) filters of the EVOS microscope. Merged image of a single cyst (DIC + calcofluor white stained cells, (**C**) and their respective individual calcofluor white (**D**) and DIC (**E**) images. Toxicity of QACs to *A. castellanii* cyst assessed using the trypan blue assay with viable (**F**) and dead (**G**) cysts without and with respectively, cytosolic blue staining. IC_50_s of cysts treated with QAC16 (closed square) and QAC18 (open square) ranging from 37.5 µg/ml to 0.07 µg/ml for 24, 48 and 72 h respectively (**H**). Data in (h) is mean ± SD; n = 4 independent experiments performed in triplicates. Student’s t-test showed significant difference between 48 h and 72 h for QAC18 relative to 24 h at p < 0.01. No significant difference was observed for QAC16 at the same duration and degree of significance.

**Figure 8:**
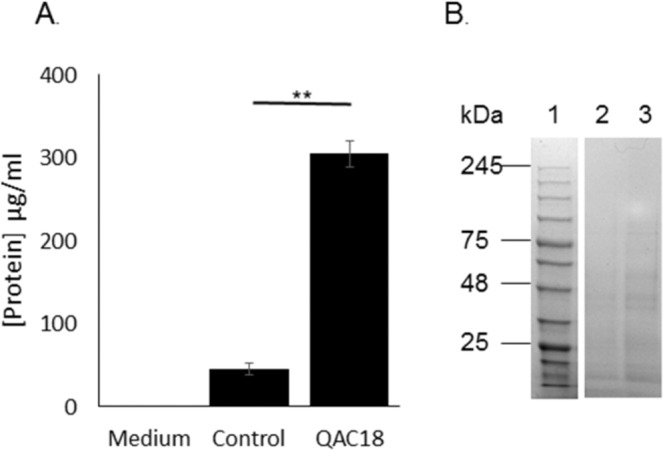
Extrusion of cytoplasmic constituents of *A. castellanii* cysts to the external milieu after QAC treatment. Protein (**A**) released after the incubation of 10^5^ *A. castellanii* cysts, with (QAC18) and without (control) QAC18 at 37.5 µg/ml for 24 h, measured in spent medium using the Bradford assay. Data is mean ± SD respectively (**A**); n = 4 independent experiments performed in triplicates. Student’s t-test showed significant difference between mean [proteins] in control and QAC18-treated experiments. **p < 0.01. (**B**) Extracellular proteins were visualised with SDS-PAGE. Key: Lane 1; Protein ladder, Lane 2; spent media form untreated cysts, Lane 3; spent medium from QAC18-treated cysts.

Lastly, we observed that QAC16 and QAC18 and not QAC12 had activity against matured *A. castellanii* cysts (calcofluor white positive and SDS-resistant (0.5%, w/v; Fig. 7A–G) and their estimated IC_50_s were 19.00 ± 0.03 µg/ml and 15.00 ± 0.06 µg/ml respectively after 96 h (Table 2). The cysticidal activity of QAC16 to QAC18 strongly correlated with (a) the alkyl-carbon chain lengths (Table 2) and the duration of cell exposure to the inhibitor (Fig. 7H). Like trophozoites, drug treatment resulted in leakage of proteins (Fig. 8A). Proteins concentrations in spent medium of QAC-treated cysts increased 6.6-fold and the extruded proteins are shown in Fig. 8B. Further, cyst viability estimated using a linked reversion-viability assay (that involved inhibitor removal, incubation of cells in PG medium alone for 7 days to induce excystation and viability estimation via Alamar blue), with QAC18 and QAC16 at 37.5 μg/ml after 24 h and 48 h respectively and for QAC18 at 18.75 μg/ml after 48 h showed no resurgence or resazurin oxidation (Table 3).

## Discussion

APCs and QACs are effective antimicrobials against fungi, bacteria, some protists and also have anti-tumour properties. In this study, we have confirmed their toxicity against *Acanthamoeba* trophozoites^34,35^, and showed that QACs were more effective than their APCs counterpart, perhaps due to the formation of larger micelles (QACs, 8.8 ± 0.8 nm; APCs, 5.8 ± 1.0 nm)^36^. Interestingly, the activities from both compound-types were strongly correlated with their alkyl-carbon chain lengths against trophozoites, a key indicator for micellar size and hydrophobicity. In addition, we noted that the duration of contact between the compound and the protist was important; longer alkyl-chain molecules required shorter contact times (<24 h) than their shorter counterpart. This is probably related to the nature of their interaction, with complementary alkyl carbon chains in the protist’s plasma membrane. Metabolomics analysis have shown that fatty acids 20–30 carbon atoms long^37^ in *A. castellanii* are abundant in its plasma membranes, presumably enough for long chain QACs to produce rapid solubilisation and death. This supports with similar study in the parasitic protist, *Leishmania*, where lipidomics analysis showed fatty acids with 18–25 alkyl-carbons long to be abundant^38^ and coincidentally, the shorter APCs and QACs were more efficacious. Previously as 1.9 × 10^10^ of QAC18 molecules have been shown to adhere to per unit surface area of the plasma membrane of *A. castellanii* trophozoites to cause leakage^39,40^. So, there is a possibility that in our study, micelles instead assembled on the surface of the cell, increased permeability and caused leakage of proteins and DNA in QAC-treated trophozoites.

Our results have also shown that it is possible that, the cationic but less so their zwitterionic APC counterpart, can induce rapid reversal of the net negative charge of the protist plasma membrane to positive, with detrimental effect as described for fungi^41,42^ and *Leishmania* spp.^43–45^. Finally, the observed DNA compacting caused by QAC18 and not QAC12 suggested that QAC had an intracellular target and offered a third death mechanism for QACs in *A. castellanii*^41,42,46–48^. Unexpectedly, death was not due to apoptosis. The data suggests that QAC18 may be complexing with compacted DNA, disrupting the normal cell cycle.

Compounds with activity against *Acanthamoba* cysts are limited. Our study has shown that the QACs used in this study have cysticydal activities and cause death via leaking, possibly emanating from conformational changes and weakening of the inter-fibre bonds of the cellulose cell wall. Evidence for this is indirect, and based on the loss of calcofluor white staining in QAC18-treated cysts. For example, the loss of the natural auto-fluorescent of carboxymethyl cellulose after QAC activity has been linked with conformational changes in cellulose^46^ and as such, QACs are being used to alter the structural conformation of wood cellulose in paper manufacturing^49^.

Further, we observed that QAC12 promoted *A. castellanii* trophozoites growth, suggesting a role as an energy substrate, as its presence increased trophozoites biomass and delayed encystation by 96 h. Interstingly, the delayed encystation of trophozoites with QAC12 made them sensitive to APC16 but less so to QAC18; possibly allowing APC16 to exert its cytotoxicity without the induction of encystation. This is evidenced by the cytostatic to cytotoxic switch of APC16 without and with QAC12, respectively. At this stage, the interaction of these mixed surfactants with different charges to lower the surface interfacial tension of the molecule, their ease of forming micelles (CMCs), molar solubilisation ratio cannot be excluded^50,51^. More investigations are required to substantiate their involvement.

Finally, we have presented sufficient evidence to show QAC18 alone and QAC12 in combination with APC16 are efficacious against *Acanthamoeba castellanii*, trophozotes and cysts, enough for consideration as a preventative management strategy for cleansing contact lens^5,32,34^. At present, cytotoxicity information of these mixed surfactants provided in this study against human epithelial and corneal cell lines are absent and as such are a significant barrier for use as a medical device. However, Polyquad, a QAC, is a preferred preservative in contact lens cleaning solutions utilised by a number of manufacturers^52^. In addition, QAC “neutralizing” agents such as β-cyclodextrin and NeutraQuat™ that bind free QACs, have very high neutralisation efficacy, such that their use as a post-sterilisation step should be suitable to minimise eye damage negating the use of tap water^53,54^. In short, we propose that combinations of different alkylphosphocholines could be introduced in disinfectant protocols as an effective preventative management protocol for contact lens care, to prevent *Acanthamoeba* contamination and protect compliant contact lens users.

## Methods

### Cell lines used in this study

*Acanthamoeba castellanii* ATCC 50370 trophozoites and cysts were cultured in Peptone Glucose (PG) medium and encystment medium^55^ respectively at 25 °C. Cysts were prepared by incubating trophozoites for 8 days in encystment medium and integrity validated with calcofluor (0.25 µg/ml) assay^56^ or sodium dodecyl sulfate (SDS, 0.5% w/v) disintegration assay^57^ and microscopic observation.

### Cytotoxicity assay

The compounds used in this study all contained alkyl-carbon chains: three APCs [dodecyl-PC (APC12), tetradecyl-PC (APC14) and hexadecyl-PC (APC16)] (Anatrace) and four QACs [docdecyl-TMAB (QAC12), tetradecyl-TMAB (QAC14), hexadecyl-TMAB (QAC16) and octadecyl-TMAB (QAC18)] (Sigma). All were prepared to a stock concentration of 1 mg/ml and diluted as required by the experimental protocol in the cytotoxicity assays. *A. castellanii* trophozoites and cysts (at 10^5^ cells/ml) with or without APCs or QACs (concentrations ranging from 150 µg/ml doubly diluted to 0.15 µg/ml to a final volume of 100 µl) in a 96-well plate were incubated at 25 °C for 96 hours.

For the combined assay, similar concentrations of QAC18 and APC16 were prepared and 18.75 µg/ml and 37.5 µg/ml of QAC12 added (to a final volume of 100 µl) were added to 10^5^ cells/ml in a 96-well plate and later incubated at 25 °C for 96 hours.

Resazurin (5 mg/ml)^58^ and trypan blue (0.4 µg/ml)^59^ were then added to trophozoites and cysts respectively after exposed to the compounds and the cell viability estimated from changes in absorbance (at 570 nm and 595 nm) and microscopically respectively.

For the drug kinetic assay, viability was assessed by the linked Alamar blue-reversion assay. Drug-treated cysts were washed with PBS and fresh PG medium was added to allow reversion to trophozoites for 5 days, after which, alamar blue was added and viability estimated as described above.

Efficacy was expressed as a percentage of untreated controls for each drug concentration used and the data used to calculate the IC_50_. Cell density was estimated using the modified Neubaur hemocytometer and expressed as cells/ml.

### Isolation of total genomic DNA

Genomic deoxynucleic acids (gDNA) from 10^6^ *A.castellanii* trophozoites (treated with and without QAC) were extracted from cells harvested by centrifugation (850 × g, 10 min), lysed with UNSET buffer (8 M urea, 150 µM NaCl, 2% SDS, 1 µM ethylenediaminetetraacetic acid (EDTA), 100 µM Tris-HCl, pH 7.5) and the DNA extracted with phenol-chloroform (1:1 v/v). The biphasic suspension with DNA enriched on the lower trizol layer was centrifuged (12,000 × g for 15 min, 4 °C) for clear separation and transferred to a new microcentrifuge tube. The DNA was precipitated with cold ethanol (100%, v/v) and 0.3 M sodium acetate, washed twice with 70% (v/v) ethanol (12,000 × g for 10 min, 4 °C), air dried and resuspended in distilled water.

### Morphometric analysis

*A. castellanii* trophozoites incubated with and without QAC at a duration determined by experimental protocol were examined microscopically and their sizes measured using the Open Laboratory (Improvision) calibration graticule and the the Holomonitor M4 live cell analyser and Holomonitor App Suite. The mean body sizes, area, volume and perimeter and the proportion of cells with a fixed interval were determined.

### Potassium (K^+^) determination assay

Potassium [K^+^] concentration in spent medium of *A. castellanii* trophozoites and cysts were harvested as described above, filtered with the 0.22 µm syringe filter and used for [K^+^] determination using the Atomic Absorption Spectrometry (ASS, absorbance 766.5 nm). A K^+^ standard curve was used to convert absorbance values to concentration (expressed as mg/ml).

### Protein and DNA determination assay

Total protein concentration in cell extract and media of QAC-treated and untreated *A. castellanii* trophozoites and cysts were estimated using the Quick Start Bradford Protein Assay (Bio-Rad) as described in the manufacturer’s instructions and the absorbances obtained at 595 nm using the Tecan Infinite M1000 Pro plate reader and expressed as ng/µl protein using a BSA standard curve. QAC18 and PG medium were used in control experiments. Proteins were visualised by SDS-PAGE gel as described previously^38^. DNA concentration in cell extract and media of QAC-treated and untreated *A. castellanii* trophozoites were estimated at 260 nm using the Nanodrop ND-1000 and expressed as ng/µl DNA. DNA was visualised with DNA agaraose gel (0.75%) as described previously^38^.

### QAC-DNA interaction assay

The interaction between the gDNA from *A. castellanii* with QAC12 or QAC18 was investigated at DNA:QAC ratios of 1:0, 1:1, 1:10 and 1:20 for 15 min and the concentration of unreacted DNA in the complex estimated at 260 nm using the Nanodrop ND-1000 at 260 nm.

### Statistical analysis

Descriptive statistics of mean and standard deviation values were used to represent data for at least four independent experiments each done in triplicate. To explore differences, between baseline and assay characteristics, T-test statistics were calculated with a statistical threshold of significance set at p < 0.01 or p < 0.05.
